# Childhood Radiation Exposure and the Adult Neoplasms that followed: A Case Report

**DOI:** 10.51894/001c.144433

**Published:** 2025-09-30

**Authors:** Madison Brown

**Affiliations:** 1 Department of Internal Medicine Henry Ford Genesys

## 70

Hodgkin lymphomas (HL) remains the most common childhood cancer in the age range of 15 -19 years old, and, as such, underscores the necessity for physicians to be aware of common late sequelae of HL treatment regimens, specifically, the increased risk for secondary malignancy.

Here we present a case of a 67-year-old female with a history of HL in childhood successfully treated with combination chemotherapy and radiation therapy who later experienced several secondary solid tumors/ malignancies including: left renal mass, uterine cancer, acoustic neuroma, adenocarcinoma of the lung, and newly diagnosed adenocarcinoma of the pancreas with metastatic liver disease. An outpatient workup found no genetic cause for her multiple malignancies and instead correlated it with her history of radiation exposure as a child. Initially hospitalized for generalized weakness, dehydration, and increased frequency of falls, all of which had begun after her third round of chemotherapy. The case was further complicated by pneumonia, lower extremity deep vein thrombosis, and a gastrointestinal bleed. CT Abdomen and Pelvis with and without contrast showed small bilateral effusions with bibasilar consolidation suggesting pneumonia, acute colitis, 3.5 cm left adnexal cyst, 2.5 cm left renal mass, and mild left renal hydronephrosis with probable 1 mm ureteral calculus. Esophagogastroduodenoscopy found a large anastomotic ulcer and gastritis, while colonoscopy showed large internal and external hemorrhoids, sigmoid colon colitis, and ascending colon polyp with removal. After an episode of respiratory distress, she was transferred to the intensive care unit and diagnosed with a pulmonary embolism.

This case report explores the correlation between childhood radiation therapy and the later risk of secondary malignancy as well as other medical complications these individuals face. Many of these patients are at an increased risk of secondary malignancies, as well as cardiovascular, endocrine, and pulmonary dysfunction. More specifically, our patient had multiple primary solid tumor malignancies throughout her lifetime, as well as hypertension, diabetes mellitus, and hypothyroidism, all common risks associated with early-age radiation therapy. Healthcare providers need to be aware of all the possible sequelae of this growing and aging population to better care for these individuals.

